# Immunity to Bee Stings

**Published:** 1898-02-26

**Authors:** 


### IMMUNITY TO BEE STINGS.

We have several times referred to the subject of snake bite and to the immunity to snake poison produced by repeated inoculation with progressively increasing doses of the venom, as illustrating the mode by which immunity to infectious diseases is in all probability produced in man. An observation lately recorded in the *Practitioner* is worthy of note in the same relation. Dr. Langer states that the beekeepers in Bohemia acquire a complete immunity to the stings of those insects, and that some seem to be immune from the first, possibly by inheritance from immunised fathers. So complete is the immunity that men, while intoxicated, have been stung in from 50 to 100 places with less resulting suffering than is felt by most persons from flea bites.

